# The impact of bariatric and metabolic surgery on cancer development

**DOI:** 10.3389/fsurg.2022.918272

**Published:** 2022-07-15

**Authors:** Fabian Lunger, Pauline Aeschbacher, Philipp C. Nett, Georgios Peros

**Affiliations:** ^1^ Department for Visceral Surgery and Medicine, Bern University Hospital, University of Bern, Bern, Switzerland; ^2^ Department of Visceral and Thoracic Surgery, Cantonal Hospital of Winterthur, Winterthur, Switzerland

**Keywords:** Bariatric surgery, cancer risk, metabolic surgery, sleeve gastectomy, roux en y gastric bypass

## Abstract

Obesity (BMI ≥ 30 kg/m^2^) with related comorbidities such as type 2 diabetes mellitus, cardiovascular disease, sleep apnea syndrome, and fatty liver disease is one of the most common preventable risk factors for cancer development worldwide. They are responsible for at least 40% of all newly diagnosed cancers, including colon, ovarian, uterine, breast, pancreatic, and esophageal cancer. Although various efforts are being made to reduce the incidence of obesity, its prevalence continues to spread in the Western world. Weight loss therapies such as lifestyle change, diets, drug therapies (GLP-1-receptor agonists) as well as bariatric and metabolic surgery are associated with an overall risk reduction of cancer. Therefore, these strategies should always be essential in therapeutical concepts in obese patients. This review discusses pre- and post-interventional aspects of bariatric and metabolic surgery and its potential benefit on cancer development in obese patients.

## Introduction

Obesity (BMI ≥ 30 kg/m^2^) has become an increasing pandemic disease in Western countries, often associated with comorbidities such as type 2 diabetes mellitus, cardiovascular disease, sleep apnea syndrome, or fatty liver disease (1). Today, almost 20% of all adults in the European Union (EU) are affected by obesity, and the prevalence is increasing yearly (2). Besides increased age, geographic and social-economic factors are considered risk factors for developing cancer such as colon, ovarian, endometrial, breast, pancreatic, and esophageal cancer (3–7). Weight loss of as little as 10% by either an increase in physical activity, diets, drug therapies (GLP-1-receptor agonists), or bariatric and metabolic surgery is reported to be associated with an overall risk reduction of cancer development (6, 8, 9). Among all weight loss therapies, bariatric and metabolic surgery is the most effective and sustainable form of obesity treatment with a long-term weight reduction of 25% (9–13) expecting a more significant effect on risk reduction of cancer development compared to non-invasive treatment methods (8, 10, 14).

This review discusses pre-and postoperative aspects of bariatric and metabolic surgery and its potential benefits on risk reduction of cancer development.

## Pathogenesis of cancer development in the obese

Besides many pathways known to affect the pathogenesis of cancer development in the obese, the molecular interactions and potential targets regarding carcinogenesis are diverse and, therefore, not yet fully understood (15). The oncologic risk and its association to the obese have been poorly studied in terms of molecular interactions, although this association has been described for decades (15, 16).

Lifestyle, including low physical activity, is known to be one major independent risk factor for both obesity and cancer development, especially in colon, endometrial, renal, and esophagus cancer (15). In colon cancer (16), strong evidence exists for the link between reduced dietary fiber intake and carcinogenesis (17–19). Furthermore, more and more information is available on the relationship between food processing and its role in cancer development (18, 19). A red meat diet is known to be correlated with both an increased risk for obesity (18) and the prevalence of colon cancer (19).

Other carcinogenesis factors include hormones such as estrogens and their metabolic precursors, mainly produced by adipose tissue (20–26). Chronic exposure to these compounds is reported to be associated with an increased risk for breast cancer in obese women (27, 28). This is, in general, more frequently seen in patients with type 2 pre-diabetes mellitus (20, 24). But not only women are affected by increased levels of estrogens and their metabolic precursors (24, 25, 29, 30). There is growing evidence that men with type 2 diabetes mellitus are also at increased risk for prostate cancer (20).

Inflammation is another essential mechanism involved in regenerative processes and seems to be in particularly involved in carcinogenesis (26, 31–36). Obesity, often associated with impaired glucose hemostasis and insulin resistance, is reported to be connected to a higher activity of inflammatory cytokines such as tumor necrosis factor (TNF)-*α* and interleukin (IL)-6 (27). Elevated levels of unbound insulin-like growth factor (IGF)-1 protein, for example, promote tumor growth by insulin and IGF-1 receptors (20, 21, 27), whereas leptin and adiponectin play another essential role in these molecular mechanisms (37). Glucagon-like peptide (GLP)-1 and its receptor have also been implicated in the insulin metabolism that plays a dominant role in cell and tumor growth (27, 35). Therefore, it is considered that GLP-1 receptor agonists, acting as a powerful weight loss drug therapy, might also play an important role in cancer development of different tumors (15, 16). This supports the concept that high levels of GLP-1 and adipokines by an expansion of adipose tissue might amplify these interactions (27). Some types of cancer, especially colon (22, 23) and breast cancer (24), are known to be associated with high levels of adipokines (21, 25, 26).

## Weight loss therapies

Increased physical activity and changes in diet are useful tools for weight loss therapy, but for some patients, these methods are not effective or only temporary (8, 10, 14). In the long-term, less than 1% of patients undergoing these conservative weight-loss therapies can reduce their BMI from 40 to 45 kg/m^2^ to below 30 kg/m^2^ (8). Medication with GLP-1 receptor agonists and/or bariatric and metabolic surgery might be an option (10, 14). On average, patients undergoing treatment with GLP-1 receptor agonists lose 15% of their weight, whereas patients undergoing bariatric surgery lose 25% within the first six to twelve months after treatment with only a small proportion of relapses (9–13). Bariatric and metabolic surgery is currently the most effective and sustainable form of weight loss therapy (8), with a significantly better effect on the remission rate of type 2 diabetes mellitus and other cardiovascular and metabolic diseases (9–13). This also impacts the risk of cancer development after weight loss therapy leading to an increased life expectancy of the treated patients (9).

## Tumor screening prior to bariatric and metabolic surgery

According to the American Association of Clinical Endocrinologists (AACE)/American College of Endocrinology (ACE), The Obesity Society, the American Society for Metabolic and Bariatric Surgery (ASMBS), the Obesity Medicine Association (OMA), and the American Society of Anesthesiologist (ASA), all these clinical guidelines propose an age- and family history-related, risk-based tumor screening before bariatric and metabolic surgery in obese patients (1, 38).

In all guidelines, a screening gastroscopy is recommended as a routine preoperative diagnostic test to assess Helicobacter pylori- and reflux esophagitis, the significant risk factor for Barrett's esophagus and esophageal cancer (38, 39). However, the absolute necessity of this examination is still under debate and remains controversial (1, 38, 40–50).

Proponents emphasize that a preoperative gastroscopy reveals not only an HP- and reflux-related esophagitis or Barrett's metaplasia, but also other asymptomatic gastrointestinal pathologies, which has a great impact on the choice of the bariatric and metabolic procedure (sleeve gastrectomy *versus* Y-Roux gastric bypass) (9–13, 39, 42–44, 48–50). While the incidence of reflux esophagitis after sleeve gastrectomy increases by about 30%, Y-Roux gastric bypass is the state-of-the-art bariatric and metabolic surgery against both obesity and reflux esophagitis (10, 12, 13, 44, 46). In addition, hiatal hernias, which are very common in patients with reflux esophagitis, can also be treated surgically simultaneously (8, 44–50).

On the other hand, a recent meta-analysis reviews how preoperative gastroscopies change the way of surgical treatment in obese patients. Besides high costs by the high frequency of bariatric and metabolic procedures worldwide, only 0.4% of these patients' surgery was delayed or canceled (38, 50).

Colonoscopy, computed tomography (CT) scans, ultrasound or other screening examinations for cancer are not routinely recommended and are rarely used because of the tremendous effort required for the patient and the associated additional health costs (1, 38). Nevertheless, these screening examinations are often non-specific, and tumors tend to be found by chance (3, 4, 6). In non-invasive weight loss therapies like diets or drug therapies, such as treatment with GLP-1-receptor agonists, these screening examinations are not even considered to use for tumor screening (4, 6). However, these obese patients tend to have the same risk of developing cancer as those undergoing bariatric and metabolic surgery (8, 44–50).

Clear criteria for a preoperative endoscopy (gastro- and colonoscopy) for specific risk groups are needed to adequately select patients benefiting from such detailed screening examinations (1, 4, 6, 8, 9, 38), and a selective endoscopy in these patients might be more appropriate in the tumor screening before bariatric and metabolic surgery (38, 41–50).

## Impact of different bariatric and metabolic procedures

More than 85% of the current bariatric and metabolic procedures worldwide consist either of sleeve gastrectomy or of Y-Roux gastric bypass (8–14, 42–49). Compared to the Y-Roux gastric bypass, the sleeve gastrectomy is a restrictive surgery that clinically reveals both restrictive and malabsorptive components and is limited to the stomach (41–43). There is no shortening or regrouping of different small intestine sections like in the Y-Roux gastric bypass (44–49). However, both sleeve gastrectomy and Y-Roux gastric bypass lead to similar entero-hormonal changes enabling a weight reduction of 25% in the long-term (10, 42–47, 49). Nevertheless, anatomical changes must be considered by the choice of type of the surgical intervention. For example, in patients with liver diseases access to the common bile duct must be preserved, which precludes performing a Y-Roux gastric bypass in such a case (41, 43, 45). A sleeve gastrectomy, on the other hand, always offers access to these anatomical structures (42–47).

Newer bariatric and metabolic procedures like single anastomosis stomach-ileal bypass with sleeve gastrectomy (SASI-S) or the single anastomosis duodenal-ileal bypass with sleeve gastrectomy (SADI-S) are considered to combine the benefits of a sleeve gastrectomy and mini-gastric bypass (omega-loop gastric bypass) while reducing the risk of malnutritional problems in the long-term (8, 10–12). Vertical banded gastroplasty, jejuno-colic, or jejuno-ileal bypasses are considered outdated procedures and are widely abandoned due to the severe malabsorptive and malnutritional long-term complications (9, 12). Adjustable gastric banding and biliopancreatic diversion are still performed in certain obese subgroups counting for less than 2% of all bariatric and metabolic procedures (11, 13, 14)

## Risk modification of cancer development after bariatric and metabolic surgery

Compared to the immediate effects of bariatric and metabolic surgery on metabolic diseases, such as type 2 diabetes mellitus, sleep apnoea syndrome, and fatty liver disease with a remission rate of 50–75% within the first weeks and months, longer and more extensive studies are needed to prove the risk reduction for cancer development (8–14, 31–34).

After bariatric and metabolic surgery, the oncological risk seems to be significantly reduced for breast cancer, endometrial, and other women-specific cancers (51). But the tumor's biology, behavior, and aggressiveness depend not only on the gender but also on the patient's age and metabolic and biological conditions (52, 53). Thus, it is unsurprising that the risk reduction for cancer development must not be examined and considered generally but under these respective aspects and groups (15, 16, 54).

### Type 2 diabetes mellitus, bariatric and metabolic surgery, and the risk of cancer development

One of the most notable and immediate effects of bariatric and metabolic surgery in type 2 diabetes mellitus patients with hyperinsulinemia and peripheral insulin resistance is the restoration of glucose hemostasis (55–62).

Based on direct mechanisms, bariatric and metabolic surgery has a tremendous impact on glycemic metabolism and substantially reduces immediately circadian blood glucose fluctuations (55, 56, 59–61). Thus, insulin therapy can be reduced or even discontinued in patients with insulin-dependent type 2 diabetes mellitus within a few days after surgery (55, 57–59, 61, 62). These effects are the same for sleeve gastrectomy and Y-Roux gastric bypass (59, 60). Data from studies with a follow-up of at least ten years confirm that bariatric and metabolic surgery significantly also impacts the risk of cancer development in type 2 diabetes mellitus patients (36–38, 54). Women with type 2 diabetes mellitus and an HbA1C ≥ 8% appear to have an increased risk of developing a tumor than men (35, 36). This might be explained by the fact that endocrine interactions between the insulin signaling and the gonadal axis leading to increased estrogen levels are thus higher in premenopausal women than in men (37, 38).

Indirect mechanisms that lead to a risk reduction for cancer development include increased postprandial secretion of satiety hormones such as GLP-1, peptide YY (PYY), and oxyntomodulin (OXM) (52, 53). The postoperatively increased GLP-1 levels and their effect on insulin are discussed as a possible mediator of angiogenesis and cell growth (20–28, 30, 33–37, 53). Accordingly, to these mechanisms, this effect is increased in insulin-dependent type 2 diabetes mellitus patients (31, 32, 34, 52–57, 62).

### The risk of colorectal cancer after bariatric and metabolic surgery

Colorectal cancer is one of the most common tumor diseases, accounting for almost 10% of all cancers worldwide, and its prevalence is increasing every year (54, 63–66). The risk of developing colorectal cancer depends on the degree of overweight and increases with higher BMI over time, which is more pronounced for colorectal cancer than for rectal cancer (66).

It is therefore surprising that studies conducted to prove this relationship showed an increased risk of colorectal cancer after bariatric and metabolic surgery. Two studies from Sweden both confirmed this unexpected finding (63, 64). In both studies, the risk of colorectal cancer was higher than in overweight non-operated patients and increased steadily with time after surgery (63, 64). An increased risk of colorectal cancer, especially after Y-Roux gastric bypass, was also demonstrated in a recent study from England, in which almost 9,000 patients after surgery were compared with non-operated overweight patients (66). This could be explained by the fact that hyperproliferation of rectal mucosal cells can be observed Y-Roux gastric bypass, which could be related to an increased risk of colorectal cancer after bariatric and metabolic surgery (66).

Nevertheless, recent cohort studies found no increased risk of colorectal cancer after bariatric and metabolic surgery compared to the general population (63, 66). In addition, a recent meta-analysis suggests that bariatric and metabolic surgery may also reduce the risk of colorectal cancer compared to non-operated obese individuals (66).

These contradictory results are certainly related to the limited data and number of studies available and highlights the need for larger prospective studies with a longer follow-up.

### Barrett metaplasia and esophageal cancer in the of context bariatric and metabolic surgery

In Barrett's metaplasia, a more extensive retrospective study showed that in 43% of patients with these preoperative conditions Y-Roux gastric bypass led to remission of acid reflux and was associated with histological regression of Barrett's metaplasia (42–45). In contrast, patients who underwent primary sleeve gastrectomy developed in one third a *de novo* Barrett's metaplasia (45). A secondary switch in these patients to Y-Roux gastric bypass resulted in a significant reduction in acid exposure and histological remission of reflux esophagitis in over 80% of these patients (42, 43). Since the introduction of sleeve gastrectomy about twenty years ago, Barrett's metaplasia and adenocarcinoma of the distal esophagus have become the focus of clinical interest, as a *de novo* incidence of more than 30% of acid reflux has been reported following primary sleeve gastrectomy (45, 46). In this context, an estimated 0.05% to 0.5% of new esophageal cancer cases worldwide are expected to occur after bariatric and metabolic surgery, particularly after sleeve gastrectomy (45, 46).

In our opinion, a comprehensive preoperative assessment of the acid exposure of the distal esophagus and esophagogastric junction using routine gastroscopy and 24-hour pH-manometry, as well as an appropriate follow-up after bariatric and metabolic surgery, especially sleeve gastrectomy, is mandatory.

## Conclusion

It has long been known that the risk for certain cancers is strongly related to obesity and that weight loss can substantially reduce this risk in the long term. Bariatric and metabolic surgery offer the most significant potential for weight loss, with an average weight loss of 25%, providing a significant post-interventional overall risk reduction for cancer development. This link acts through direct and indirect mechanisms whose molecular levels are very complex and not yet fully understood.

Preoperative tumor screening before bariatric and metabolic surgery is mainly based on recommendations of different societies rather than solid evidence. However, a thorough clinical evaluation, including detailed family history, is highly recommended and helps to find the proper bariatric and metabolic procedure which fits the patient's prerequisites and needs. Since no tumor screening is usually carried out during conservative or medical weight loss therapy, more attention should be paid to this point in managing overweight patients in the future.

Regarding a preoperative gastroscopy or 24-hour pH-manometry, we believe this screening method is a low-risk intervention and should be performed when planning bariatric and metabolic surgery, especially since these patients require close follow-up anyway. However, significantly more data are needed to make a more detailed and precise recommendation which is suitable for practice.

Looking at the two most often performed bariatric and metabolic procedures worldwide in more detail, Y-Roux gastric bypass, compared to sleeve gastrectomy, seems to be more effective in patients with Barrett's metaplasia by an almost complete remission of acid reflux. This also means that patients after a primary sleeve gastrectomy to reduce weight could also benefit from a Y-Roux gastric bypass as a secondary procedure to treat or to avoid *de novo* acid reflux and Barrett's metaplasia.

Although weight loss therapies, including bariatric and metabolic surgery, cannot be routinely recommended as a cancer prevention strategy, considerations in this context should always be made in treating obese patients.
